# *Fasciola gigantica* Cathepsin L1H: High Sensitivity and Specificity of Immunochromatographic Strip Test for Antibody Detection

**DOI:** 10.3390/tropicalmed8030164

**Published:** 2023-03-11

**Authors:** Phawiya Suksomboon, Pornanan Kueakhai, Narin Changklungmoa

**Affiliations:** 1 Faculty of Allied Health Sciences, Burapha University, Long-Hard Bangsaen Road, Mueang District, Chonburi 20131, Thailand; phuean_ppb@hotmail.com (P.S.); pornanan@go.buu.ac.th (P.K.); 2 Research Unit for Vaccine and Diagnosis of Parasitic Diseases, Burapha University, Long-Hard Bangsaen Road, Mueang District, Chonburi 20131, Thailand

**Keywords:** *Fasciola gigantica*, cathepsin L1H, immunochromatographic strip test

## Abstract

Fasciolosis is a zoonotic disease caused by *Fasciola gigantica* or *F. hepatica* infections, which are frequently occurring parasites in animals and humans. The present gold-standard diagnostic technique involves finding parasite eggs through microscopy. However, this method is also restricted due to low specificity and low sensitivity. An alternative to coprological diagnosis is the immunochromatographic strip (ICS) test, which is rapid, simple, convenient, and cost-effective, with high sensitivity and high specificity. Cathepsin L1H (CathL1H) is a cysteine protease secreted by *F. gigantica*, which is found in high amounts in newly excysted juvenile (NEJ) and juvenile stages. Cathepsin L1H plays an important role in both the immune response to invading pathogens and in the ability of some pathogens to evade the host immune system. The present study aims to develop an ICS test and detect antibodies against CathL1H in mice and cattle serum using the recombinant *F. gigantica* Cathepsin L1H (rFgCathL1H) and rabbit anti-rFgCathL1H antibody. The *F. gigantica*-infected serum and non-infected serum of mice and cattle were tested using the ICS test. Moreover, the strip results were confirmed with an indirect enzyme-linked immunosorbent assay (indirect ELISA). The relative sensitivity, specificity, and accuracy of the ICS strip were 97.5, 99.99, and 99.00%, respectively. Therefore, these data suggest that the ICS method could be used to detect *F. gigantica* antibodies to highly enhance throughput, reduce costs, and determine the best alternative on-site method.

## 1. Introduction

Some of the world’s major public health problems are various zoonotic diseases or neglected tropical disease. Fasciolosis is a harmful disease across several continents. The two species commonly referred to as liver flukes and implicated in fasciolosis are *F. hepatica* and *F. gigantica* [1]. *Fasciola* spp. is a leading cause of illness in humans [2] and ruminants [3] on the cattle, buffalo, and sheep, due to accidentally ingested contaminated aquatic vegetation or water. The infection of both species has direct effects, such as the wellbeing of human populations and economic burden of livestock, especially regarding the decreasing level of milk and meat production [4], poor food conversion, weight gain, impaired fertility, anemia, hypoproteinemia, and meat production. Fasciolosis is highly endemic and reported in many parts of the world, including America, Europe, Oceania [5], East Africa [6], South America, and north, west, and southeast Asia, including Vietnam and Thailand [7]. *F. gigantica* is an extraordinary species primarily found in Thailand, Laos, and Cambodia.

Currently, coprological analysis or microscopic examination such as the Kato–Katz [8] is unreliable and cannot differentiate *F. gigantica* eggs from morphologically identical eggs from *Fasciolopsis* spp. [9] and *Echinostoma* spp. [10]. Moreover, this method randomly collects specimens from feces, which may result in parasite eggs being missed. Furthermore, *Fasciola* spp. become sexually mature and release eggs 3–4 months after infection. Almost 10 weeks after infection, parasite eggs can be detected in the stool. However, the accumulation of parasite eggs in the gallbladder is lower; thus, it difficult to detect parasites’ eggs in feces. The polymerase chain reaction (PCR) assay has been used to detect the DNA of *F. hepatica* in the feces of infected sheep [11]. However, PCR needs high technical skills, sensitive equipment, a sterile environment, and long preparation times, and it is difficult to run the process in rural areas. Sandwich enzyme-linked immunosorbent assay (sandwich ELISA) and indirect ELISA are commonly used for detecting antigens or antibodies. These were developed for the detection of *F. gigantica.* The disadvantages of sandwich ELISA and indirect ELISA are that they are more complex, time-consuming, require specialized equipment and reagents, and are more expensive. Thus, a new alternative rapid and accurate diagnostic tool is necessary to develop for laboratory testing and field-based testing, namely, the immunochromatographic strip (ICS) test. The ICS test is a one-step assay that can be performed quickly and easily, making it more suitable for field or point-of-care settings where a rapid result is needed.

The classical laboratory diagnosis of a fasciolosis is based on microscopic examination, PCR, and ELISA to detect an antigen of *Fasciola* spp. or antibody in serum or milk [12]. Antibodies against fasciolosis in human can be detected two weeks post-infection, before the presence of eggs in the feces [13]. Recently, a sandwich ELISA was developed to detect *F. hepatica* Cathepsin L1 in human serum with a sensitivity and specificity of 97% and 100%, respectively [13]. A previous study developed an ELISA assay to detect the *F. hepatica* antibody in cattle serum [14]. The diagnostic sensitivity and specificity of the ELISA test were 98% and 96%, respectively. Hence, laboratory diagnosis is not suitable for detection under certain conditions. The development of a diagnosis technique should be designed to be rapid, easy to use, accurate, visible to the naked eye, and have high sensitivity and specificity. Currently, an ICS test has been applied for detecting viruses [15] and parasites [16]. The colloidal gold-labeled antigen or antibody is most commonly used as a tracer. The result can be seen within 15–20 min. However, the ICS test method has not been developed to detect antibody for fasciolosis.

*Fasciola* spp. secrete several different antigens, which are fatty acid binding proteins (FABP), Glutathione S-transferase (GST), Leucine aminopeptidase (LAP), Saposin-like protein 2 (SAP2), and Paramyocin. However, only Cathepsin L is abundant during all stages of the parasite’s development. Many studies agree that the major enzyme component secreted by *F. gigantica* is Cathepsin L [17]. Cathepsin L is a type of protease enzyme that is found in a variety of organisms, including the parasitic flatworm *F. gigantica*. In *F. gigantica*, cathepsin L has been shown to play a role in the digestive process and in the degradation of host tissues during infection. Previous studies have demonstrated that *Fasciola* spp. secreted enzymes that were predominantly cysteine proteinases [18], which are believed to be involved in the parasite’s survival and play a necessary role in the biology of parasitic organisms. Cathepsin L1H was highly expressed in the epithelial cells of the digestive tract in the infective stage, juvenile stage, and immature and mature *Fasciola* spp. [19]. The level of Cathepsin L and Cathepsin B in the somatic NEJs stage of *F. hepatica* has been found to be 25.71%. Other proteinases found in NEJ excretory and secretory (ES) products have been identified as belonging to the Cathepsin L family, which was the most abundant secreted group (50.15%), followed by Cathepsin B (25.11%), Legumain (24.70%) and metalloproteinases (0.04%) [20]. In addition, adult ES products have been shown in the same direction as NEJ E/S products, which is Cathepsin L (44.74%), followed by Cathepsins B (26%) and Legumains (15%) [20]. Infection by *F. gigantica* results in high production of Cathepsin L1H, which stimulates the host’s immune system to produce antibodies, and as a result, the circulating antibodies against *Fasciola* spp. can be detected as early as 2–4 weeks [2]. Thus, Cathepsin L1H has been proposed as an important target for developing more sensitive and specific diagnostics to detect antibodies in the serum of the parasite’s early and adult stages. In this study, rFgCathL1H was developed to conjugate colloidal gold, which can detect anti-FgCathL1H antibodies in serum. The objective of the present study was to develop and evaluate the ICS test using the rFgcathL1H and rabbit anti-rFgCathL1H antibody for rapid antibody detection. Its utility makes it a powerful tool for diagnosis.

## 2. Materials and Methods

### 2.1. Expression and Purification of Recombinant F. gigantica Cathepsin L1H (rFgCathL1H)

The FgCathL1H nucleotide sequence (GenBank accession number AY428949.1) [19] was inserted to pET30b vector. The pET30b-rFgCathL1H was expressed in *Escherichia coli* BL21(DE3). Expression was facilitated by adding 0.5 mM isopropyl-B-D-1-thiogalactoside (IPTG) (Calbiochem, Merck, Darmstadt, Germany) to 1 mM final concentration at 37 °C. The rFgCathL1H was purified by nickel-nitrilotriacetic acid (Ni-NTA) affinity chromatography (QIAGEN, Hilden, Germany). The supernatant was loaded into column at 4 °C. The column was washed twice with a wash buffer at 4 °C. The rFgCathL1H protein was eluted with elution buffer. All fractions and the eluted protein of rFgCathL1H were collected and were analyzed by sodium dodecyl sulfate-polyacrylamide gel electrophoresis (SDS-PAGE). The rFgCathL1H was thawed and dialyzed by 0.01 M PBS, pH 7.4 at 4 °C overnight. The concentration of rFgCathL1H was determined using Lowry’s method and stored at −20 °C until needed for further experiments.

### 2.2. Preparation and Purification of Rabbit anti-rFgCathL1H

The rabbit anti-rFgCathL1H used in this study was obtained from the Research Unit for Vaccine and Diagnosis of Parasitic Diseases, Burapha University. One rabbit was injected with rFgCathL1H to produce polyclonal the antibody anti-rFgCathL1H. All procedures were conducted under ethical principles and guidelines for the use of animals and were approved by Institutional Animal Care and Use Committee (IACUC), Burapha University. Briefly, rFgCathL1H was mixed with complete Freund’s adjuvant for prime. Additionally, rFgCathL1H was mixed with incomplete Freund’s adjuvant for the first, second, and third boosts. The rabbit was injected subcutaneously with rFgCathL1H. The rabbit polyclonal antibody (PoAb) anti-rFgCathL1H was purified using an Econo-Column chromatography column with Hi-bind™ Protein G-Agarose (Biovision, Milpitas, CA, USA). The IgG was eluted by elution buffer (0.1 M citric acid, pH 2.75). The elute was collected and neutralized immediately with a neutralization buffer (1 M Tris-HCl, pH 9.0). The elute was examined by SDS-PAGE. The elution of the IgG fraction was dialyzed by 0.01 M PBS, pH 7.4 at 4 °C, 4 times, for 12 h each. The purified rabbit anti-rFgCathL1H dialysis was transferred to a Ultracel-3K, (Amicon ^®^ Ultra Millipore Corporation, Billerica, MA, USA) at 4500 rpm at 4 °C. The rabbit anti-rFgCathL1H concentration was determined by Lowry’s method and analyzed by SDS-PAGE.

### 2.3. Serum Sample from F. gigantica-Infected Mice and Cattle

A total of 100 male mice from the National Laboratory Animal Center, Mahidol University, Nakorn Pathom, Thailand, were divided into 2 groups: 40 infected mice and 60 non-infected mice. In the infected group, fifteen metacercaria were fed to mice. Then, mice were terminated 4 weeks post-infection, and serum was collected from mice infected with *F. gigantica*. Additionally, collected serum from non-infected mice was used as the negative control.

Twenty-one cattle serum samples were kept from the slaughterhouse in Phetchaburi, Thailand, where all cattle presented parasite infections: *Cotylophoron cotylophorum*, *Fischoederius cobboldi*, *Gastrothylax crumenifer*, *Haemonchus placei*, and *Paramphistomum cervi*. The observation of *Fasciola* spp. infection in the liver confirmed the presence of infected serum. Cattle were divided into 2 groups: 10 non-infected cattle (non-*Fasciola* spp. infection) and 11 infected cattle (*Fasciola* spp. infection).

### 2.4. Detection of the Antibody against FgCathL1H by Indirect ELISA

A 96-well microtiter ELISA plate (Thermo Scientific, Roskilde, Denmark) was used, and each well was coated with 50 µL /well of 2 ug/mL rFgCathL1H protein and incubated overnight at 4 °C. The coated plate was washed 3 times with phosphate-buffered saline with Tween™ 20 (PBS-T) (0.05% Tween 20 in PBS). The coated plate was blocked with 100 µL/well of 1% bovine serum albumin (BSA) (Capricorn Scientific, Ebsdorfergrund, Hessen, Germany) in PBS at 25 °C for 1 h. The infected mouse serum and non-infected mouse serum (negative sample) diluted in PBS, pH 7.4 (1:8) were added and incubated at 25 °C for 1 h. The goat anti-mouse IgG conjugated HRP (Southern Biotech, Birmingham, AL, USA) was diluted at 1: 5000 in PBS, and pH 7.4 was added to the 50 µL/well and incubated at 25 °C for 1 h. Finally, 3,3′,5,5′-Tetramethylbenzidine (TMB) (SeraCare Life Sciences, Milford, MA, USA) solution was added to the 50 µL/well and incubated in a dark room at 25 °C for 15 min. 1N HCl halted the reaction, and the plate was immediately read using a SpectraMax^®^ ABS microplate reader at OD_450_.

### 2.5. Optimization Concentration and pH of Colloidal Gold-Conjugated rFgCathL1H

The optimal concentration of rFgCathL1H for conjugation with the colloidal gold was determined. Briefly, the colloidal gold (DCNovations, Moorestown, NJ, USA) was adjusted to pH 7.0 with 0.1 M NaOH. The rFgCathL1H was diluted with 0.1 M phosphate buffer, pH 7.0 to a final concentration of 0 mg/mL (Negative control), 0.03125 mg/mL, 0.0625 mg/mL, 0.125 mg/mL, 0.25 mg/mL, 0.5 mg/mL, 0.6 mg/mL, 0.7 mg/mL, and 0.8 mg/mL. The rFgCathL1H solution of each concentration was added into 1 mL of colloidal gold solution (pH 7.0). Subsequently, the rFgCathL1H and colloidal gold were gently rotated for 30 min at 25 °C. Then, 50 µL of 10% NaCl was added and rotated for 10 min. A total of 10% NaCl can cause the aggregation of gold nanoparticles by disrupting the electric double layer and causing the particles to come together. The color was noted after 2 h. The result was read by a SpectraMax^®^ ABS microplate reader at OD_525_ absorbance. The minimum concentration that remained red and did not change color was collected as the optimum concentration.

The rFgCathL1H solution (0.5 mg/mL) was prepared using a 0.1 M phosphate buffer in difference pH 5.5, pH 6.0, pH 6.5, pH 7.0, pH 8.0, and pH 9.0. Each solution was added to 0.5 mL of colloidal gold solution at the same pH and rotated for 30 min at 25 °C. After that, 50 µL of 10% NaCl was added and rotated for 10 min at 25 °C. Each pH value reaction mixture was measured using a SpectraMax^®^ ABS microplate reader at OD_525._

The appropriate concentration and pH was chosen at 0.5 mg/mL and prepared for the rFgCathL1H conjugate with colloidal gold. The rFgCathL1H 0.5 mg/mL in 0.1 M phosphate buffer (pH 7.0) was added to the colloidal gold solution (pH 7.0) and rotated for 30 min at 25 °C. After that, 10% (*w*/*v*) BSA (Capricorn Scientific, Ebsdorfergrund, Hessen, Germany) was added and rotated for 20 min at 25 °C. The mixture was centrifuged for 30 min at 10,000 rpm at 4 °C to remove residual supernatant. The gold precipitates or lower layer was collected, and passive gold diluent was added (0.1 M phosphate buffer (pH 7.0), 2% *w*/*v* sucrose, 0.05% sodium azide) to 20 OD. The gold-labeled rFgCathL1H was stored at 4 °C until further use.

### 2.6. Preparation of an Immunochromatographic Strip (ICS) Test for Antibody Detection

**Figure 1:**
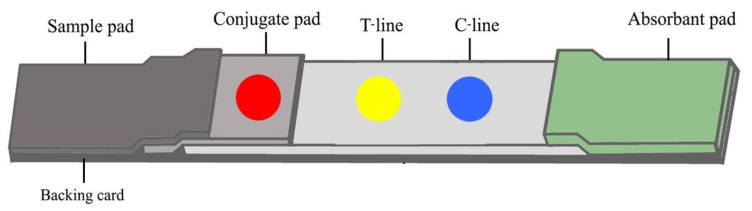
Schematic view of colloidal gold test strip for antibody detection. The strip was an assembly on polystyrene backing card. The strip consists of a sample pad, conjugate pad, nitrocellulose membrane, and absorbent pad. The conjugate pad was immobilized with rFgCathL1H-conjugated colloidal gold (**red dot**). The nitrocellulose membrane strip consists of rFgCathL1H (**yellow dot**) and rabbit anti-rFgCathL1H (**blue dot**), which were immobilized as a T-line and a C-line, respectively.

After colloidal gold conjugation with a rFgCathL1H, a test strip was prepared and assembled. Briefly, 4 µL of rFgCathL1H conjugated colloidal gold was placed on a piece of a conjugate pad (STD17) (GE Healthcare Life sciences Whatman^TM^, Buckinghamshire, UK) sized 0.4 × 0.8 cm and dried in laminar at 37 °C for 2 h. The test line (T-line) was dispensed on a nitrocellulose (NC) membrane (AE99, GE Healthcare Life sciences Whatman^TM^, Buckinghamshire, UK) with 0.4 µL of rFgCathL1H (0.5 mg/mL), whereas 0.4 µL of rabbit anti-rFgCathL1H (2 mg/mL) served as the control line (C-line), respectively. Next, the nitrocellulose membrane was blocked with 2.5% *w*/*v* BSA (Capricorn Scientific, Ebsdorfergrund, Hessen, Germany) and diluted in 0.1 M phosphate buffer, pH 7.0, for 30 min at 25 °C. Subsequently, 5% *w*/*v* sucrose diluted in 0.1 M phosphate buffer, pH 7.0, was added on the nitrocellulose membrane for 15 min at 25 °C. After drying for 40 min at 37 °C, The ICS test were assembled onto the backing card using four components, as shown in Figure 1: a sample pad (Fusion 5), a conjugate pad (STD17), a nitrocellulose membrane (AE99), and an absorbent pad (CF7). Each part overlapped by 0.2 cm. The strip was cut into individual strips with a width of 4.0 mm/strip with a strip cutter. The strip was sealed and kept at room temperature until used.

### 2.7. Construction and Calculation Result of ICS Test for Antibody Detection

The ICS test system detected an antibody in the serum, as shown in Figure 1. The purified rFgCathL1H conjugated colloidal gold (0.5 mg/mL) was placed on a conjugate pad (STD17). The conjugate pad (STD17) was cut into 0.4 × 0.8 mm. The nitrocellulose membrane (AE99) was used for the T-line and C-line. The purified rFgCathL1H (0.5 mg/mL) and rabbit anti-rFgCath1H (2 mg/mL) were dispensed onto a nitrocellulose membrane (AE99) as T-line and C-line, respectively. The nitrocellulose membrane (AE99) was cut into 0.4 × 2.5 mm. The sample pad (fusion 5) and absorbent pad (CF7) were cut into 0.4 × 1.7 mm. All parts were assembled and kept at room temperature.

Each serum sample was tested in duplicate and expressed as an individual mean OD. All data from the detection of antibody against FgCathL1H in sera of mice experimentally infected with *F. gigantica* were calculated and analyzed using the standard diagnostic indices, including sensitivity, specificity, accuracy, positive predictive values (PPV), negative predictive values (NPV), false-positive rate, and false-negative rate, which were calculated using the Galen method, as shown in the following formulae: sensitivity (%) = [no. of true positives/(no. of true positives + no. of false negatives)] × 100; specificity (%) = [no. of true negatives/(no. of true negatives + no. of false positives)] × 100; PPV = [no. of true positives/(no. of true positives + no. of false positives)] × 100; NPV = [no. of true negatives/(no. of true negatives + no. of false negatives)] × 100; accuracy = [all with true positives and negatives/all tests done] × 100; false positive rate (%) = [no. of false positives/(no. of false positives + no. of true negatives)] × 100; and false-negative rate (%) = [no. of false negatives/(no. of false negatives + no. of true positives)] × 100.

## 3. Results

### 3.1. Expression and Purification of Recombinant Protein FgCathL1H

**Figure 2:**
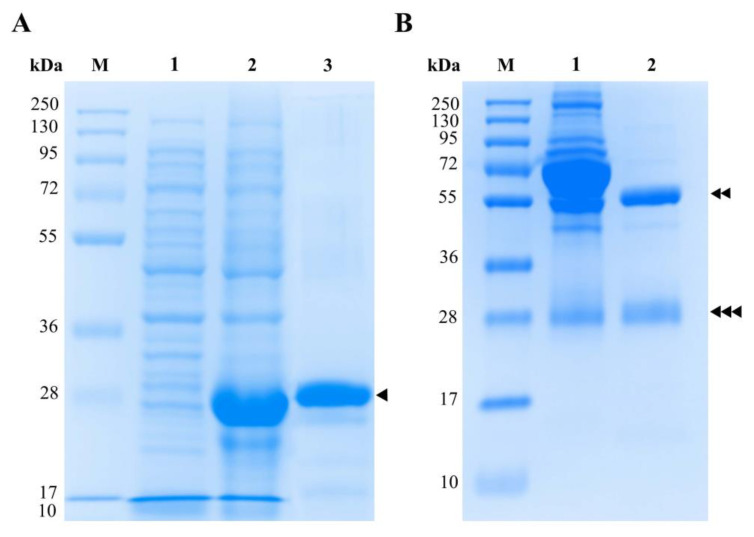
The 12.5% SDS-PAGE analysis of rFgCathL1H expression (**A**) and purified rabbit anti-rFgCathL1H (**B**), followed by Coomassie brilliant blue staining. M is a molecular size protein marker. In (**A**), lane 1 is non-induction, lane 2 is the induction with IPTG, and the purification of rFgCathL1H in lane 3 is approximately 28 kDa (single-head arrow). In (**B**), lane 1 is rabbit anti-rFgCathL1H serum (containing all serum proteins including PoAb anti-rFgCathL1H). Lane 2 is the purified IgG of rabbit anti-rFgCathL1H using Protein G. The expected molecular weight of heavy chain and light chain at 55 kDa and 25 kDa, as shown by the double-head arrow and triple-head arrow, respectively.

The pET30b-rFgCathL1H gene transformed into competent *E. coli* BL21 (DE3). The protein was expressed when induced with IPTG at a final concentration of 1 mM at 4 h. Then, the purified rFgCathL1H was analyzed by 12.5% gel SDS-PAGE. The SDS-PAGE results qualitatively demonstrate that the molecular weight of rFgCathL1H was 28 kDa (Figure 2A).

### 3.2. Purification of Rabbit anti-rFgCathL1H Serum

The rabbit anti-rFgCathL1H serum was collected and purified by Hi-bind^TM^ Protein G agarose. The eluted fraction of rabbit anti-rFgCathL1H IgG was analyzed 12.5% gel SDS-PAGE. After purification, the polyclonal antibody bands formed were indicated by two bands, a heavy chain and a light chain, at approximately 55.0 kDa and 25.0 kDa, respectively (Figure 2B).

### 3.3. Optimization Concentration and pH of Colloidal Gold-Conjugated rFgCathL1H

**Figure 3:**
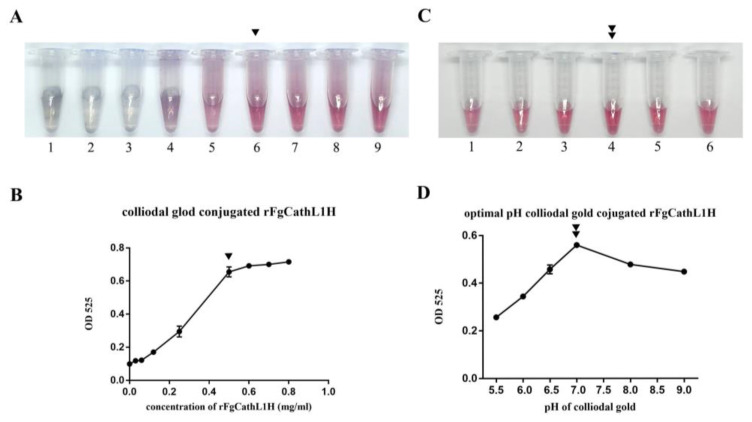
Determination of optimum concentration and pH value of rFgCathL1H conjugated colloidal gold. Photograph of the different concentrations of rFgCathL1H. The color of the rFgCathL1H-conjugated gold solution changed color from red to clear when the concentration was not appropriate. In Figure (**A**), the optimal concentration of rFgCathL1H marked with colloidal gold (1) 0 mg/mL, (2) 0.03125 mg/mL, (3) 0.0625 mg/mL, (4) 0.125 mg/mL, (5) 0.25 mg/mL, (6) 0.5 mg/mL, (7) 0.6 mg/mL, (8) 0.7 mg/mL, and (9) 0.8 mg/mL, from left to right. In Figure (**B**), determining the optimal ratio of colloidal gold and rFgCathL1H for conjugation. The graph showed a curve of the absorbance at 525 nm. The curves of OD_525_ values represented the appropriate concentration at 0.5 mg/mL (single-head arrow). In Figure (**C**), the photograph shows the different pH of colloidal gold reagent that conjugated with rFgCathL1H at 0.5 mg/mL and was read by a SpectraMax^®^ ABS microplate reader at OD_525_ (1) pH 5.5, (2) pH 6.0, (3) pH 6.5, (4) pH 7.0, (5) pH 8.0, and (6) pH 9.0. In Figure (**D**), the graph shows the curve of the absorbance at 525 nm and depicts the appropriate pH 7.0 (double-head arrow).

After 10% of NaCl was added to each tube of different concentrations (0 mg/mL, 0.03125 mg/mL, 0.0625 mg/mL, 0.125 mg/mL, 0.25 mg/mL, 0.5 mg/mL, 0.6 mg/mL, 0.7 mg/mL, and 0.8 mg/mL), the color was observed by the naked eye and photographed within 15–20 min. The low antigen concentration (0, 0.03125, 0.0625, and 0.125 mg/mL) changed from red to colorless or purple (tube 1–4), as shown in Figure 3A, whereas 0.25, 0.5, 0.6, 0.7, and 0.8 mg/mL (tube 5–9), as shown in Figure 3A, did not change color because the exceeded protein antigen was conjugated with colloidal gold. The lowest concentration of rFgCathL1H was suitable at 0.5 mg/mL and OD_525_ was 0.655, as can be seen in Figure 3B. Therefore, 0.5 mg/mL of rFgCathL1H was collected to conjugate with the colloidal gold solution and immobilize on a conjugate pad.

In this study, it can be seen that the colloidal gold solutions of different pH values were adjusted by 0.1M NaOH as pH 5.5, 6.0, 6.5, 7.0, 8.0, and 9.0 (tube 1–6), respectively, as shown in Figure 3C. A suitable pH was observed, and no color change after the addition of 10% NaCl was noted. The absorbance results of the pH value of colloidal gold solution marking rFgCathL1H was measured by a SpectraMax^®^ ABS microplate reader at 525 nm, and the results are shown in Figure 3D. When the pH reached 7.0, the OD_525_ was at saturation point at 0.55. Therefore, the optimal pH of the colloidal gold is 9.0 (Figure 3D).

### 3.4. Detection of the Antibody against FgCathL1H by Indirect ELISA

**Figure 4:**
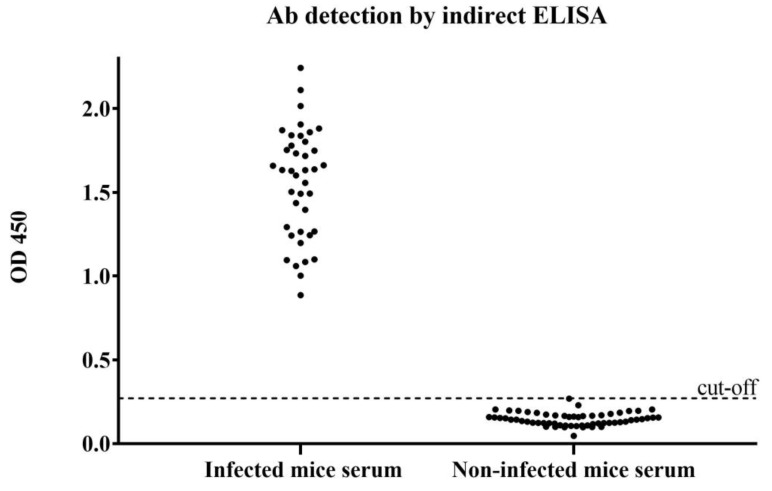
Detection of anti-rFgCathL1H antibody in the sera of mice. Mice were infected with *F. gigantica* (*n* = 40), and non-infected mouse serum (*n* = 60) was classified as positive and negative by indirect ELISA assay.

Mice were infected with *F. gigantica* serum (*n* = 40), and non-infected mouse serum (*n* = 60) was tested using an indirect ELISA assay. The results were confirmed with the ICS test. As can be seen from Figure 4, when rFgCathL1H coated 96 microplate wells, infected mouse serum was detected and showed high OD values when compared with non-infected mouse serum. The range OD values of four-weeks-post-infected mouse serum and non-infected mouse serum were 0.886–2.243 and 0.046–0.268, respectively. The cut-off OD value of infected mouse serum samples was 0.270, so an OD sample with an OD value > 0.270 was considered to be a positive result.

### 3.5. Development of Rapid Antibody Detection Kits for Fasciolosis

**Figure 5:**
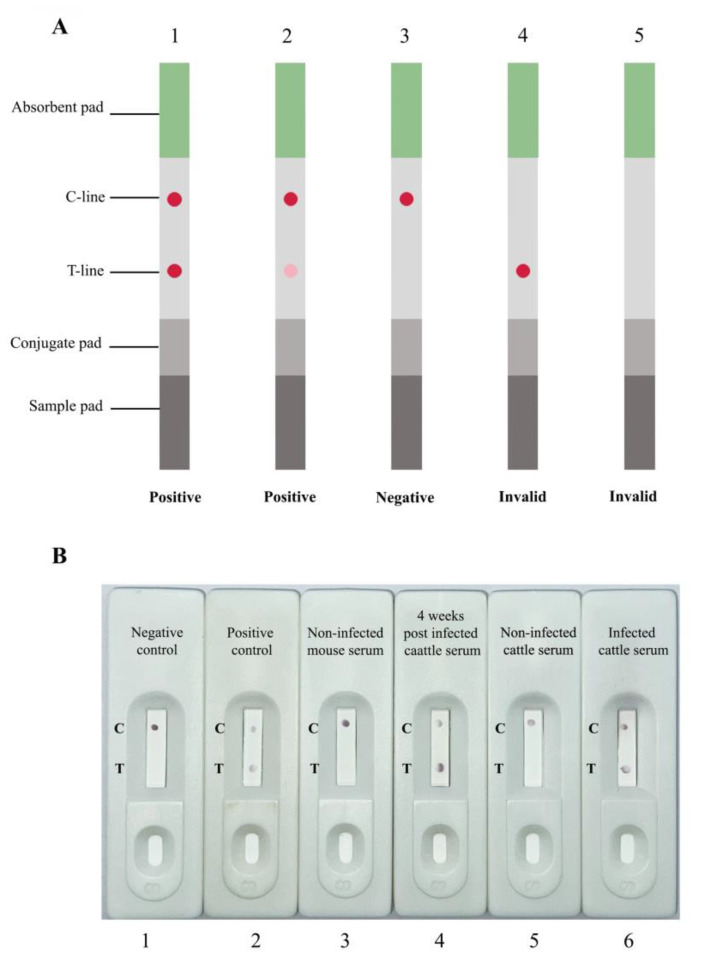
(**A**) Schematic illustration result of colloidal gold test strip. (A1 and A2) Positive test, (A3) negative test, and (A4 and A5) invalid. (**B**) Representative photos of rapid antibody detection assay. (1) A test strip was tested by using 0.1 M phosphate buffer, pH 7.0, which was interpreted as negative. (2) The sample was a purified rabbit anti-rFgCathL1H concentration 1 mg/mL as a positive control. The samples blood of (3) non-infected mouse serum, (4) four-weeks-post-infected mouse serum administration, (5) non-infected cattle serum, and (6) infected cattle serum was tested by ICS test. If the T-line appeared within 15 min, the result was positive for the presence of anti-rFgCathL1H antibodies.

All samples were tested in order to detect anti-rFgCathL1H IgG antibodies. A total of 15 µL of serum samples was mixed with 120 µL of 0.1 M phosphate buffer, pH 7.0. The serum was placed on the sample pad. The mixture of serum and buffer was released onto the absorbent pad. Antibodies in serum specific to antigen targets developed color on the T-line and the C-line. After the reaction, the immunochromatography strip test was taken photo within 15 min. The results of the ICS test were read, as illustrated in Figure 5A. The results were read as negative if no red dot appeared on the T-line. Positive cases were identified if red dots appeared on the T-line and C-line. However, if no red dot appeared on the C-line, the test was invalid.

### 3.6. Immunochromatographic Strip (ICS) Test for Antibody Detection

In this study, infected mouse serum, non-infected mouse serum, naturally infected cattle serum, and non-infected cattle serum were tested for antibody detection using an ICS test. The results are shown in Figure 5B. The 15 μL serum sample was mixed with 120 μL of 0.1 M phosphate buffer, pH 7.0, and applied to the sample pad on a cassette. All infected serum samples of mice and cattle were represented with a red dot on C-line and T-line as a positive result. This result indicated that anti-rFgCathL1H antibodies had been found in the serum. All cassettes of the ICS test were recorded after 15 min. In addition, the antibody detection of non-infected mice serum samples and infected mice serum was determined by indirect ELISA assay, which was confirmed by the ICS test (Table 1). Notably, 40 positive sera were tested, which were shown as positive by indirect ELISA. A total of thirty-nine sera were positive, while one tested negative when using the ICS test. A total of 60 negative sera were also found when analyzed using the indirect ELISA and ICS tests. A total of 21 cattle sera were tested by ICS test to detect antibodies against FgCathL1H. The results showed that 11 infected *F. gigantica* cattle sera revealed positive results and 10 non-infected cattle serum were negative when using the ICS test (Table 2).

### 3.7. Sensitivity and Specificity of ICS Test

The diagnostic sensitivity, specificity, PPV, NPV, accuracy, false-positive rate, and false-negative rate of the ICS test are shown in Table 3. The sensitivity of the ICS test was 97.5% for 40 mice serum samples. In addition, the specificity was 99.99% for 60 non-infected mice serum samples. The PPV, NPV, and accuracy were 99.99%, 98.36%, and 99%, respectively. The false-positive rate and false-negative rate were 0% and 2.5%, respectively.

## 4. Discussion

There are several disadvantages regarding the current diagnostic methods for fasciolosis, including low sensitivity, which easily leads to false negative results, and thus, repeat stool examinations may be required. Additionally, a parasite’s eggs may not be present in the feces until 12–14 weeks after infection, which can further complicate diagnosis. In the acute phase, eggs are likely to be missed during routine stool screening. However, antibodies against the *Fasciola* spp. can be detected 2–4 weeks after the initial infection [2], and hence, antibody/serological tests could be the new and appropriate method. This would move toward the development of a suitable method for *Fasciola* spp. in the future. A lateral flow test for the serodiagnosis of human fascioliasis was described in another report [21]. This test was designed to detect antibodies against the recombinant *F. hepatica* cathepsin L1 protein, which is associated with the disease. The test employs protein A and MM3 monoclonal antibodies as detector reagents in the test and control lines, respectively. These antibodies can bind to both cathepsin L1 and L2 proteases [21]. Thus, antibody tests can be used to determine the prevalence of a disease in a population, which can inform public health decisions, such as vaccination campaigns and quarantine measures. Accordingly, in this study, an ICS test with high sensitivity, specificity, and accuracy was developed for rapid antibody detection in fasciolosis.

The present study indicates that antibody anti-*Fasciola gigantica* cathepsin L1H (FgCatL1H) in infected mouse and cattle serum can be detected by rFgCathL1H using an ICS test. It is possible that this ability of FgCatL1H could be developed and used as a diagnostic tool, as FgCathL1H is expressed in all life stages of *F. gigantica* and found in other trematodes [19,22]. Moreover, antibody anti-FgCatL1H can be easily detected in infected serum due to a highly immunogenic host. Thus, it is possible that this ability of FgCatL1H could be used for the diagnosis of early and chronic phases. Moreover, ICS testing is considered to be a tool to detect antigens [23,24] or antibodies [25] in medical field, comprising a test strip for the detection of an antigen or antibody in sample solutions, such as a secretion, serum, stool samples, and urine. The ICS test strip has been extensively applied for the diagnosis of viruses or parasites, such as the laryngotracheitis virus [26], the SARS-CoV-2 virus [27], *Schistosoma japonicum* [16,28], *Angiostrongylus cantonensis* [29], *Toxoplasma gondii* [30,31], *Leishmania donovani* [32], and *F. gigantica* [33]. However, no ICS tests for detecting fasciolosis using rFgCathL1H antigens have been developed to detect anti-FgCathL1H antibodies. In this study, rFgCathL1H was developed to detect antibody anti-FgCathL1H in infected mouse and cattle serum through ICS tests. Additionally, Xifeng et al. produced recombinant Cathepsin L1D and developed an ICS test for detecting *F. hepatica*-specific antibodies [34]. The *Staphylococcal aureus* protein A complex was used as a conjugate with gold nanoparticles. The T-lines and C-lines were labeled as rCatL1D protein and sheep IgG, respectively. The specificity and sensitivity of the ICS test were 100% and 97%, respectively [34]. In our study, we used rFgCathL1H conjugated with colloidal gold, as it can be more specific to antibodies than protein A in infected animal serum. In previous research, Wang et al. applied *F. gigantica* excretory-secretory products (FgESP) to detect antibodies in mice serum present at the T-line. Goat anti-mouse secondary antibodies coated the C-line [33]. However, testing infected cattle serum requires the changing of the protein at the C-line to anti-buffalo secondary antibody 2G7 [33]. In our study, we purified rabbit anti-rFgCahL1H, which is able to attach itself to both the antibodies of mice and cattle. Our results showed that the sensitivity, specificity, and accuracy of the ICS strip were 97.5, 99.99, and 99.00%, respectively.

One of the most important factors is the optimal concentration of rFgCathL1H and pH. All these parameters need to be optimized before assembling the test kits. In the concentration test, the concentration of rFgCathL1H was chosen at 0.5 mg/mL to conjugate with colloidal gold. It was observed that when the lower concentrate of rFgCathL1H 0–0.125 mg/mL changed color to purple or became colorless if concentration was not appropriate (Figure 3A). The properties of NaCl involved Na^+^ and Cl^−^ ions attacking the surface electric potential of the colloidal gold [35]. The effect of 10% NaCl surrounded by colloidal gold particles reduces the energy barrier. The whole gold nanoparticle surface is covered with rFgCathL1H, so after adding 10% NaCl, the low concentration of rFgCathL1H leads to agglomeration during the preparation of the solution. Additionally, 10% NaCl was added, and the color in different pHs was observed. In the range of pH 5.5–9.0, pH 7.0 showed the maximum value of OD 525 (Figure 3C) and was considered to be suitable for conjugating colloidal gold and rFgCathL1H. Our study successfully developed a method of detecting antibodies through the ICS test using rFgCathL1H and rabbit anti-rFgCathL1H.

## 5. Conclusions

In summary, the concentration of rFgCathL1H was chosen to be conjugated with the colloidal gold solution at 0.5 mg/mL. The suitable pH value of the colloidal gold solution was found to be 7.0. The positive results of the ICS test were confirmed by indirect ELISA. As a result, high sensitivity, specificity, and accuracy were found at 97.5, 99.99, and 99%, respectively. We efficiently developed and optimized an ICS test to detect antibody anti-rFgCathL1H in mouse and cattle serum. ICS tests can be read visually and are able to be simply interpretated, while ELISA requires an instrument to read. Furthermore, this method demonstrated great potential as a powerful tool. It could also be further developed as an innovative method that is highly effective in the diagnosis of fasciolosis. This tool could also reduce the mortality rate of the population and reduce the cost of importing test kits from abroad. As reported above, an ICS test has several advantages, such as: it is easy to use in field; there is no need for an expert; it is a portable and rapid test; and the result can be reported within 15 min. Thus, a suitable method for *Fasciola* spp. diagnosis should be developed in the future in order to establish a reliable, simple, and rapid diagnostic tool to reduce outbreak problems and for the proper diagnosis of this zoonotic infection.

## Figures and Tables

**Table 1:** Determination of antibodies in non-infected and infected mice serum using indirect ELISA and ICS test.

Method		Indirect ELISA	
		Positive	Negative
ICS Test	Positive	39	0
	Negative	1	60
	Total	40	60

**Table 2:** Determination of antibodies in non-infected and infected cattle serum using ICS test.

Method		ICS Test	
		Positive	Negative
Cattle serum	Positive	11	0
	Negative	0	10
	Total	11	10

**Table 3:** Diagnostic values of the immunochromatographic strip test for fasciolosis detection.

Diagnostic Valves	Ab Detection by ICS Test
Sensitivity (%)	97.50
Specificity (%)	99.99
Positive predictive value (%)	99.99
Negative predictive value (%)	98.36
Accuracy (%)	99.00
False-positive rate (%)	0.00
False-negative rate (%)	2.50

## Data Availability

Not applicable.
